# Supplementary material for: Plasma proteomics for biomarker discovery in childhood tuberculosis
Source: Nat Commun. 2025 Jul 19;16:6657. doi: 10.1038/s41467-025-61515-5 (PMC12276249; doi:10.1038/s41467-025-61515-5)
Supplement: Supplementary file 1 — Supplementary Information [file 41467_2025_61515_MOESM1_ESM.pdf]

SUPPLEMENTARY INFORMATION

Plasma proteomics for biomarker discovery in childhood tuberculosis

SUPPLEMENTARY NOTES

COMBO Study consortium contributors

Andrea Fossati[1,2,3], Peter Wambi[4], Devan Jaganath[5,6], Roger Calderon[7], Robert Castro[5,8], Alexander Mohapatra[5,9], Justin McKetney[1,2,3], Juaneta Luiz[10,11], Rutuja Nerurkar[5,6], Esin Nkereuwem[12], Molly F. Franke[13], Zaynab Mousavian[14,15], Jeffrey M. Collins[16], George B. Sigal[17], Mark R. Segal[18], Beate Kampman[12,19], Eric Wobudeya[4], Adithya Cattamanchi[5,20], Joel D. Ernst[5,9], Heather J. Zar[10], Danielle L. Swaney[1,2,3], Cynthia Baard[10], Yekiwe Hlombe[10], Lesley Workman[10], Margaretha Prins[10], Abdoulie Tunkara[12], Binta Saidy[12], Francis S. Mendy[12], Madikoi Danso[12], Marie P. Gomez[12], Martina Boakarie[12], Sarjo Koita[12], Sheriff Kandeh[12], Jascent Nakafero[21], Gertrude Nanyonga[21], Juliet Namboowa[21], Winnie Nabakka[21], Nsereko Moses[21], Ainebyona Aggrey[21], Alfred Andama[21], Moorine sekadde[21], Mary Mudiope[21], Ezekiel Mupere[21], Hellen Aanyu Tukamuhebwa[21]

1. J. David Gladstone Institutes, San Francisco, California,USA
2. Quantitative Biosciences Institute (QBI), University of California San Francisco, San Francisco, California,USA
3. Department of Cellular and Molecular Pharmacology, University of California San Francisco, San Francisco, California, USA
4. Uganda Tuberculosis Implementation Research Consortium, Walimu, Kololo, Kampala,Uganda.
5. Institute for Global Health Sciences, Center for Tuberculosis, University of California San Francisco, San Francisco, USA
6. Department of Pediatrics, Division of Pediatric Infectious Diseases, University of California San Francisco, San Francisco, USA
7. Advanced Research and Health, Lima, Peru
8. Department of Medicine, Division of Pulmonary and Critical Care Medicine, University of California San Francisco, San Francisco, USA
9. Department of Medicine, Division of Experimental Medicine, University of California San Francisco, San Francisco, California 94143, United States
10. Department of Pediatrics and Child Health, South African Medical Research Council Unit on Child and Adolescent Health, University of Cape Town, Cape Town, South Africa
11. Department of Pediatrics, Dora Nginza Hospital, Gqeberha, South Africa
12. Vaccines and Immunity Theme, MRC Unit The Gambia at the London School of Hygiene and Tropical Medicine, Fajara, The Gambia
13. Department of Global Health and Social Medicine, Harvard Medical School, Boston, MA, USA
14. Division of Infectious Diseases, Department of Medicine Solna, Karolinska Institutet, Stockholm, Sweden
15. Department of Global Health, Rollins School of Public Health, Emory University, Atlanta, GA, USA
16. Division of Infectious Diseases, Department of Medicine, Emory University School of Medicine, Atlanta, USA
17. Meso Scale Diagnostics, LLC., Rockville, USA
18. Department of Epidemiology and Biostatistics, University of California San Francisco, San Francisco, USA
19. Charité Center for Global Health, Charité Universitätsmedizin Berlin, Berlin, Germany
20. Division of Pulmonary Diseases and Critical Care Medicine, Department of Medicine, University of California Irvine, Irvine, USA
21. World Alliance for Lung and Intensive Care Medicine in Uganda (WALIMU), Uganda

DESCRIPTION OF ADDITIONAL SUPPLEMENTARY FILES

Supplementary Data 1. Clinical information table with clinical features and sample IDs.
Supplementary Data 2. Full proteomics output matrix of protein abundance by sample IDs.
Supplementary Data 3. Quantitative comparisons of Confirmed vs Unlikely TB.

SUPPLEMENTARY FIGURES

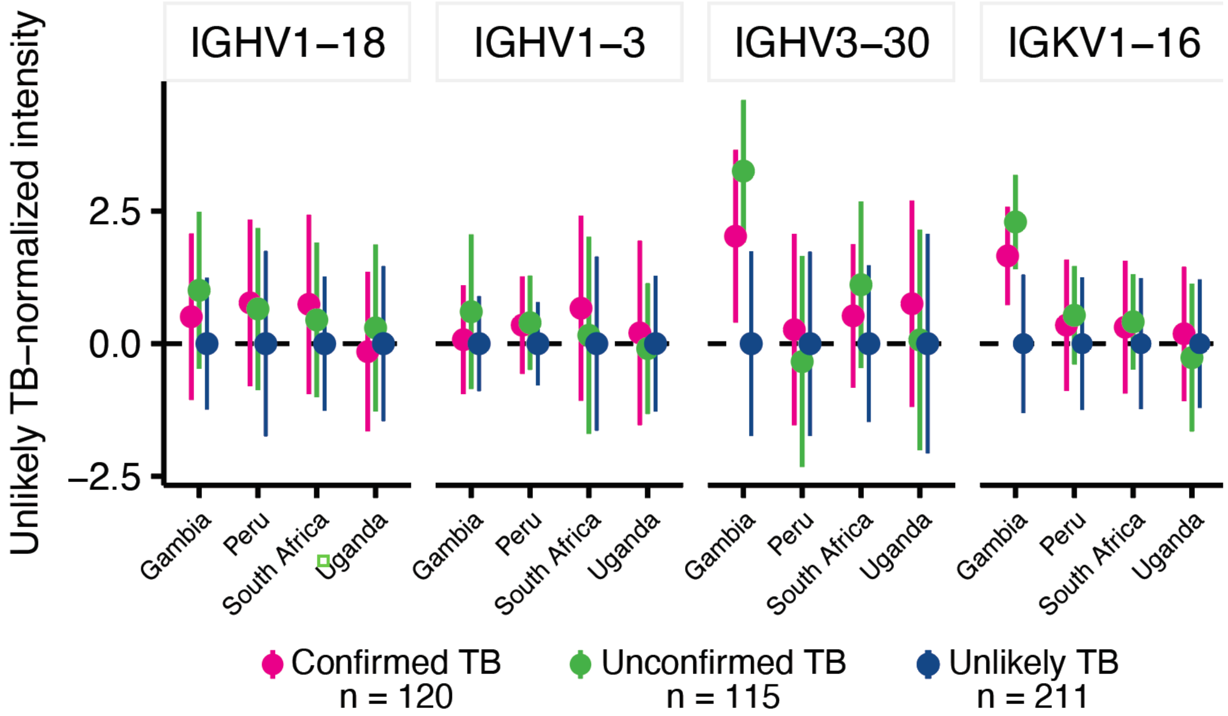

Supplementary Figure 1. Upregulated IGg level, expressed as protein intensity normalized to the global amount of the Unlikely TB condition (y-axis) stratified by clinical site (x-axis). Dots represent the mean, while lines show 1.5 x interquartile range (IQR). N-values represent individual patients.

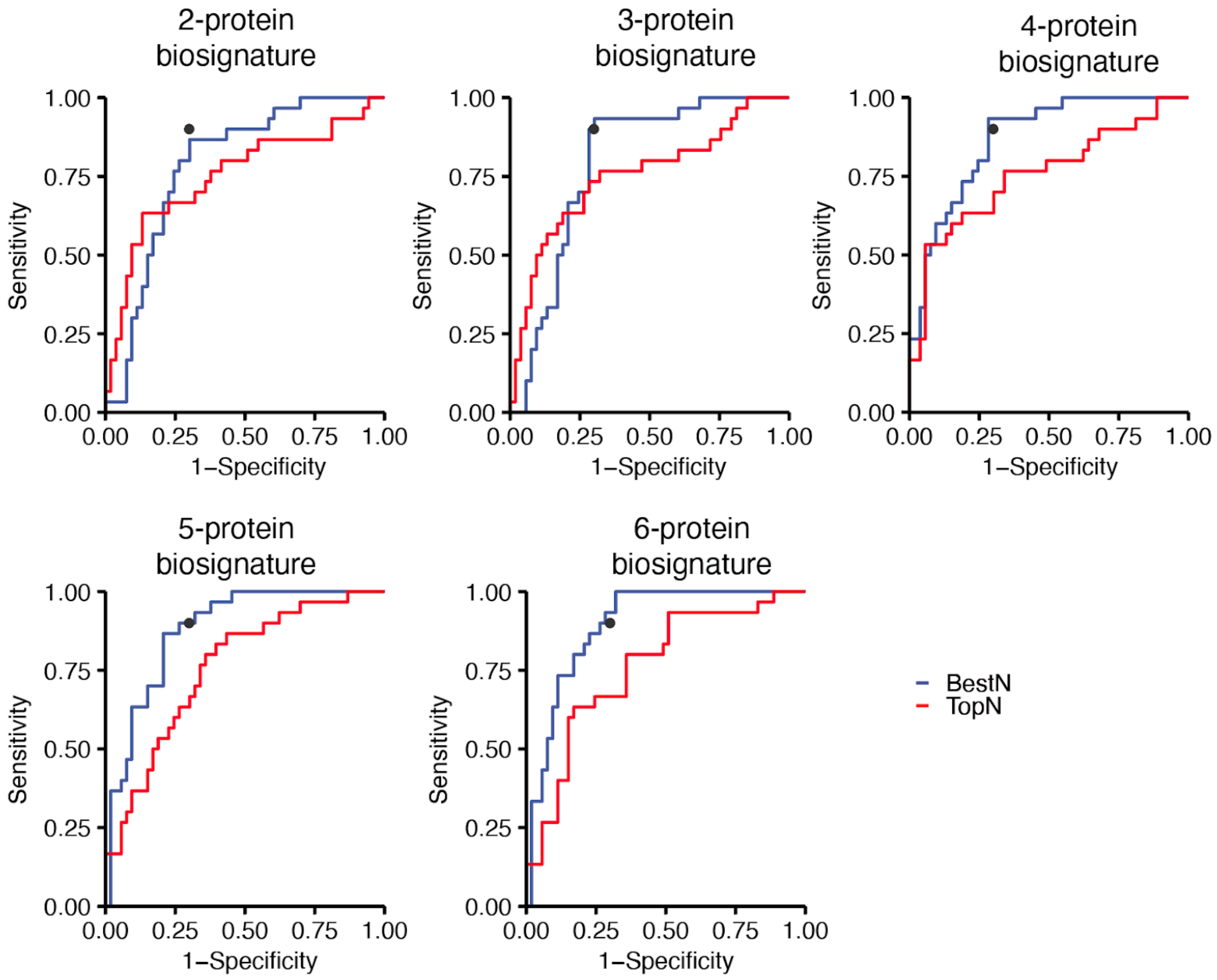

Supplementary Figure 2. ROC Curves for various numbers of features comparing the best combination (BestN, blue line) to the corresponding number of most important ones from the LASSO feature importance (TopN, red line).

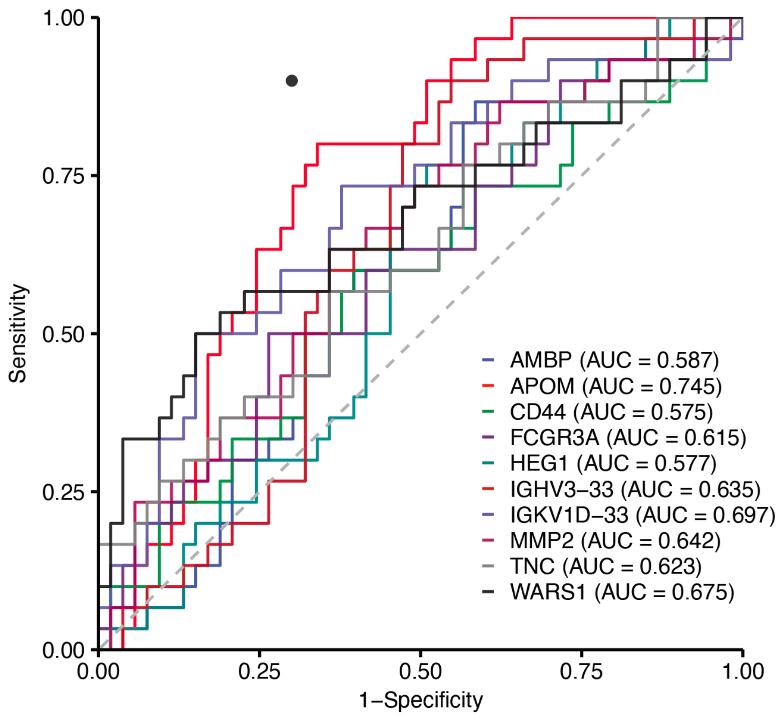

Supplementary Figure 3. Individual ROC curves for the proteins from the developed biosignatures. The black circle designates the WHO target product profile sensitivity and specificity.

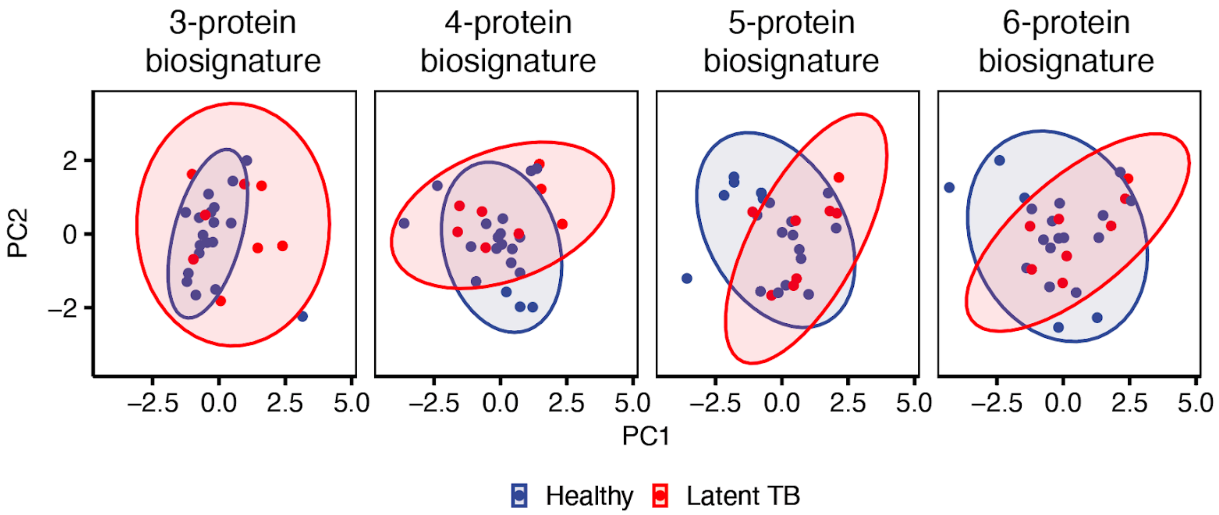

Supplementary Figure 4. Principal component analysis of healthy (n=19, blue) and latent TB (n=8, red) utilizing the derived biosignatures. X-axis shows the first component and y-axis shows the second component. Shading approximates the 95% confidence region for the 2D normal distribution of each group.

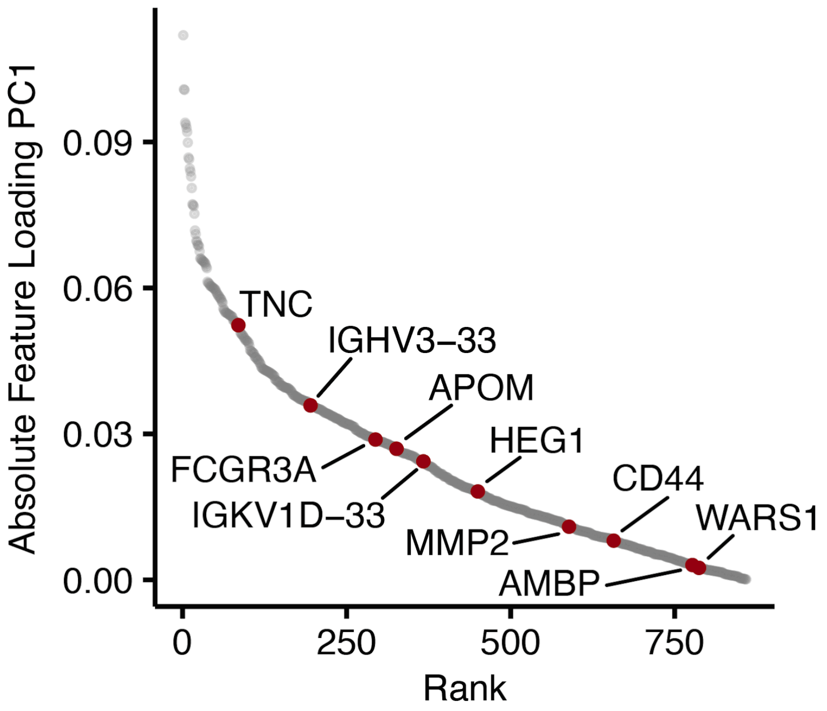

Supplementary Figure 5. Rank Plot showing the contribution of each gene to the separation in the first component between confirmed + unconfirmed TB (all models). Y-axis shows the feature loadings in absolute value and x-axis displays the corresponding rank. Biosignature proteins are highlighted in red.
