# Supplementary material for: Plasma proteomics for biomarker discovery in childhood tuberculosis
Source: Nat Commun. 2025 Jul 19;16:6657. doi: 10.1038/s41467-025-61515-5 (PMC12276249; doi:10.1038/s41467-025-61515-5)
Supplement: Supplementary file 2 — Description of Additional Supplementary Files [file 41467_2025_61515_MOESM2_ESM.pdf]

Title: Supplementary Data 1. Clinical information.

Description: A list of each sample analyzed with their corresponding metadata and clinical features.

Title: Supplementary Data 2. Proteomic abundances.

Description: A list of the individual log2 transformed protein abundance values in each sample.

Title: Supplementary Data 3. Quantitative comparisons of Confirmed vs Unlikely TB.

Description: Log2 fold change of protein abundances and the result of statistical test result for the quantitative comparison between Confirmed and Unlikely TB patients.
