# Supplementary material for: Plasma proteomics for biomarker discovery in childhood tuberculosis
Source: Nat Commun. 2025 Jul 19;16:6657. doi: 10.1038/s41467-025-61515-5 (PMC12276249; doi:10.1038/s41467-025-61515-5)
Supplement: Supplementary file 6 — Reporting Summary [file 41467_2025_61515_MOESM6_ESM.pdf]

Corresponding author(s): Heather Zar and Danielle Swaney
Last updated by author(s): 6/6/2025

Reporting Summary

Nature Portfolio wishes to improve the reproducibility of the work that we publish. This form provides structure for consistency and transparency in reporting. For further information on Nature Portfolio policies, see our Editorial Policies and the Editorial Policy Checklist.

Statistics

For all statistical analyses, confirm that the following items are present in the figure legend, table legend, main text, or Methods section.

n/a	Confirmed	
☐	☒	The exact sample size (*n*) for each experimental group/condition, given as a discrete number and unit of measurement
☒	☐	A statement on whether measurements were taken from distinct samples or whether the same sample was measured repeatedly
☐	☒	The statistical test(s) used AND whether they are one- or two-sided<br>*Only common tests should be described solely by name; describe more complex techniques in the Methods section.*
☐	☒	A description of all covariates tested
☐	☒	A description of any assumptions or corrections, such as tests of normality and adjustment for multiple comparisons
☐	☒	A full description of the statistical parameters including central tendency (e.g. means) or other basic estimates (e.g. regression coefficient) AND variation (e.g. standard deviation) or associated estimates of uncertainty (e.g. confidence intervals)
☐	☒	For null hypothesis testing, the test statistic (e.g. *F*, *t*, *r*) with confidence intervals, effect sizes, degrees of freedom and *P* value noted<br>*Give P values as exact values whenever suitable.*
☒	☐	For Bayesian analysis, information on the choice of priors and Markov chain Monte Carlo settings
☒	☐	For hierarchical and complex designs, identification of the appropriate level for tests and full reporting of outcomes
☒	☐	Estimates of effect sizes (e.g. Cohen's *d*, Pearson's *r*), indicating how they were calculated

Our web collection on statistics for biologists contains articles on many of the points above.

Software and code

Policy information about availability of computer code

Data collection	No additional software was used.
Data analysis	Raw proteomics data was analyzed with either MSFragger (v1.8, DDA data) or with DIA-NN (v1.8, DIA data), and the generated DDA library and our previous reported plasma library were merged using easypqp (https://github.com/grosenberger/easypqp). For data processing, model training, and figure generation we used the following packages in Python (v 3.8.2): scikit-learn (v1.5.1), pandas (v2.2.2), numpy (v.1.26.4), pyCombat (v),https://github.com/epigenelabs/pyComBat), joblib (v.1.4.2), seaborn (0.13.2), matplotlib (v.3.9.2), matplotlib-base (v3.9.2), scipy (v 1.13.1), statsmodel (v 0.14.2). The following packages in R (v.4.3.1, release 'Beagle Scouts') were used for figure generation: ggplot2 (v.3.5.1), RcolorBrewer (v1.1.3), viridis (v0.6.5), ggpubr (v0.6.0) , ggsci (v3.2.0). Additionally, the GSAR R package using the log2FC between Confirmed and Unlikely TB. All code for data analysis, imputation, and figure plots is available here: https://github.com/anfoss/ COMBO_code.git.

For manuscripts utilizing custom algorithms or software that are central to the research but not yet described in published literature, software must be made available to editors and reviewers. We strongly encourage code deposition in a community repository (e.g. GitHub). See the Nature Portfolio guidelines for submitting code & software for further information.

Data

Policy information about availability of data

All manuscripts must include a data availability statement. This statement should provide the following information, where applicable:

- Accession codes, unique identifiers, or web links for publicly available datasets
- A description of any restrictions on data availability
- For clinical datasets or third party data, please ensure that the statement adheres to our policy

The supporting MS data is available via the MassIVE repository with Dataset Identifier: MSV000096394, Reviewer Username: MSV000096394_reviewer, and Reviewer Password: H6DVzA8T79W1RC6c. Source Data are provided with this paper.

Research involving human participants, their data, or biological material

Policy information about studies with human participants or human data. See also policy information about sex, gender (identity/presentation), and sexual orientation and race, ethnicity and racism.

Reporting on sex and gender	We report the gender of participants, by self-report or by their caregiver. The analyses applies to all participants regardless of gender, and was not considered in the design as gender is not a major predictor of TB disease in young children. We did though assess if there was imbalance in gender across TB status and there was not. Gender is included in the source data, which can be provided in de-identified form.
Reporting on race, ethnicity, or other socially relevant groupings	We did not collect data on race or ethnicity, though have collected data on country and included this variable as part of batch correction.
Population characteristics	We analyzed plasma samples that were collected from children less than 15 years old evaluated for pulmonary TB who were previously enrolled as part of prospective diagnostic cohort studies in the Gambia, Peru, South Africa, and Uganda. Children were included if they had signs and symptoms of pulmonary TB, and excluded if they were already taking treatment for TB infection or disease for more than 72 hours
Recruitment	While recruitment and enrollment was consecutive in the parent cohort, we utilized banked frozen samples for this analysis in a random 1:1:2 ratio of Confirmed: Unconfirmed:Unlikely TB. This could have created selection bias, which we sought to mitigate with the random selection.
Ethics oversight	The studies were approved by the Mulago Hospital Ethics Research Committee, Gambian Government and MRC joint ethics committee, London School of Hygiene and Tropical Medicine, Institutional Ethics Committee for Research of National Institute of Health - Peru, University of Cape Town, and the University of California, San Francisco (UCSF) IRB.

Note that full information on the approval of the study protocol must also be provided in the manuscript.

Field-specific reporting

Please select the one below that is the best fit for your research. If you are not sure, read the appropriate sections before making your selection.

☒ Life sciences ☐ Behavioural & social sciences ☐ Ecological, evolutionary & environmental sciences

For a reference copy of the document with all sections, see nature.com/documents/nr-reporting-summary-flat.pdf

Life sciences study design

All studies must disclose on these points even when the disclosure is negative.

Sample size	We randomly selected plasma samples in a 1:1:2 ratio of Confirmed:Unconfirmed:Unlikely TB, and sample size was determined by availability of specimens and to ensure adequate precision in the test set. With a sample size of 500, and 25% held for the test set, we would be powered to measure a sensitivity of 90% +/- 12% and specificity of 70% +/-10% when comparing Confirmed to Unlikely TB.
Data exclusions	Samples were excluded if the number of peptides was below 3 standard deviations of the median number of peptides (2591), which removes samples with less than 1700 peptides.
Replication	All samples were analyzed once with the exception of selected samples where there was evidence of instrument performance deviation, including the observation of significant mass shifts or consecutive injections with reduced signal. Data for these samples was re-collected, and this re-collected data is presented in this study.
Randomization	Samples were batched by country, and randomized within a given sample preparation plate and data acquisition for each country.
Blinding	Staff were blinded to TB status during data acquisition.

Reporting for specific materials, systems and methods

We require information from authors about some types of materials, experimental systems and methods used in many studies. Here, indicate whether each material, system or method listed is relevant to your study. If you are not sure if a list item applies to your research, read the appropriate section before selecting a response.

Materials & experimental systems

n/a	Involved in the study
☒	☐ Antibodies
☒	☐ Eukaryotic cell lines
☒	☐ Palaeontology and archaeology
☒	☐ Animals and other organisms
☐	☒ Clinical data
☒	☐ Dual use research of concern
☒	☐ Plants

Methods

n/a	Involved in the study
☒	☐ ChIP-seq
☒	☐ Flow cytometry
☒	☐ MRI-based neuroimaging

Clinical data

Policy information about clinical studies

All manuscripts should comply with the ICMJE guidelines for publication of clinical research and a completed CONSORT checklist must be included with all submissions.

Clinical trial registration	Not applicable, this is not a clinical trial
Study protocol	Not applicable, this is not a clinical trial
Data collection	Not applicable, this is not a clinical trial
Outcomes	Not applicable, this is not a clinical trial

Plants

Seed stocks	*Report on the source of all seed stocks or other plant material used. If applicable, state the seed stock centre and catalogue number. If plant specimens were collected from the field, describe the collection location, date and sampling procedures.*
Novel plant genotypes	*Describe the methods by which all novel plant genotypes were produced. This includes those generated by transgenic approaches, gene editing, chemical/radiation-based mutagenesis and hybridization. For transgenic lines, describe the transformation method, the number of independent lines analyzed and the generation upon which experiments were performed. For gene-edited lines, describe the editor used, the endogenous sequence targeted for editing, the targeting guide RNA sequence (if applicable) and how the editor was applied.*
Authentication	*Describe any authentication procedures for each seed stock used or novel genotype generated. Describe any experiments used to assess the effect of a mutation and, where applicable, how potential secondary effects (e.g. second site T-DNA insertions, mosiacism, off-target gene editing) were examined.*
